# Insight on Solution Plasma in Aqueous Solution and Their Application in Modification of Chitin and Chitosan

**DOI:** 10.3390/ijms22094308

**Published:** 2021-04-21

**Authors:** Chayanaphat Chokradjaroen, Jiangqi Niu, Gasidit Panomsuwan, Nagahiro Saito

**Affiliations:** 1 Department of Chemical Systems Engineering, Graduate School of Engineering, Nagoya University, Nagoya 464-8603, Japan; eig@sp.material.nagoya-u.ac.jp (C.C.); niu@sp.material.nagoya-u.ac.jp (J.N.); 2 Department of Materials Engineering, Faculty of Engineering, Kasetsart University, Bangkok 10900, Thailand; gasidit.p@ku.ac.th; 3 Conjoint Research Laboratory in Nagoya University, Shinshu University, Nagoya 464-8603, Japan; 4 Open Innovation Platform with Enterprises, Research Institute and Academia (OPERA), Japan Science and Technology Corporation (JST), Nagoya 464-8603, Japan; 5 Strategic International Collaborative Research Program (SICORP), Japan Science and Technology Corporation (JST), Nagoya 464-8603, Japan

**Keywords:** solution plasma process, aqueous solutions, chitin, chitosan, degradation, deacetylation

## Abstract

Sustainability and environmental concerns have persuaded researchers to explore renewable materials, such as nature-derived polysaccharides, and add value by changing chemical structures with the aim to possess specific properties, like biological properties. Meanwhile, finding methods and strategies that can lower hazardous chemicals, simplify production steps, reduce time consumption, and acquire high-purified products is an important task that requires attention. To break through these issues, electrical discharging in aqueous solutions at atmospheric pressure and room temperature, referred to as the “solution plasma process”, has been introduced as a novel process for modification of nature-derived polysaccharides like chitin and chitosan. This review reveals insight into the electrical discharge in aqueous solutions and scientific progress on their application in a modification of chitin and chitosan, including degradation and deacetylation. The influencing parameters in the plasma process are intensively explained in order to provide a guideline for the modification of not only chitin and chitosan but also other nature-derived polysaccharides, aiming to address economic aspects and environmental concerns.

## 1. Introduction

**Figure 1:**
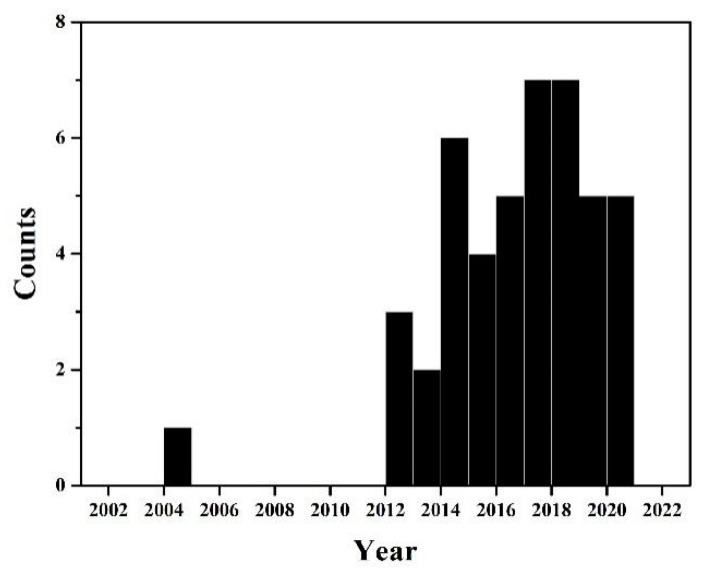
The number of articles relating to the modification of chitin and chitosan by liquid-phase plasma (data are shown in Supplementary Materials).

In physics and chemistry, plasma is fundamentally defined as one of the four states of matter. Solid, liquid, and gas states are more common on the earth due to the atmospheric condition, whereas the plasma state dominantly exists in the universe (>99%) (e.g., the sun, nebulae, etc.) [1]. Nevertheless, plasma can be artificially formed in the earth by giving sufficient thermal or electric energy supply, which ionizes the neutral gases to a quasi-neutral ionized gas [2]. The ionized gas is composed of negative and positive ions, free electrons, excited molecules, and excited atoms and molecules, as well as the emission of ultraviolet (UV) and high electric field [3]. Artificial plasma is classified as (i) thermal equilibrium plasma and (ii) non-thermal equilibrium plasma [4,5]. Thermal equilibrium plasma can be generated by a strong electrical power and, usually, under gas pressure of more than 5 kPa. Its gas and electron temperatures are nearly equal. On the other hand, in non-thermal equilibrium plasma, the gas temperature is lower than the electron temperature [4]. Non-thermal equilibrium plasma can be induced by giving sufficient energy under a vacuum system; however, it can also be generated under an atmospheric pressure environment by applying transient electrical or electrostatic discharges. Consequently, the non-thermal equilibrium plasma has offered several potential applications. Artificial plasmas in the gas phase are predominantly investigated under a wide range of operating pressures, including both vacuum and atmospheric conditions, and temperatures. A variety of gases (e.g., Ar, N_2_, O_2_, H_2_, and mixtures) is applied to generate the plasmas depending on the purposes [6,7,8]. Even though plasma is theoretically mentioned as ionized gases, plasma can be in solid form (i.e., the formation of plasmon which is induced by the collective oscillation of free electrons moving around a lattice point) and liquid form (i.e., the generation of plasma in liquid, such as a pulsed electrical discharge in liquids) [4,9,10]. In the same manner, as the plasmas in the gas phase, plasma can be directly generated in the liquid phase by providing a sufficiently high electric field on the electrodes, leading to the electric breakdown of liquids. Over the past several years, the liquid-phase plasmas have been developed, along with the gas-phase plasmas. The liquid-phase plasmas have been mainly focused as a technology for wastewater treatment and water purification, owing to their ability to induce an effective production of reactive species, for example, hydroxyl radical (^•^OH), superoxide anion (O_2_^–^), and hydrogen peroxide (H_2_O_2_) [4,11,12]. These reactive species can strongly oxidize and decompose organic pollutions and bacteria in water [12,13]. Recently, as the plasma technology being developed, the liquid-phase plasmas have been expanded and utilized for the synthesis and modification of various materials, such as noble-metal nanoparticles [13,14], metal oxides [15,16], carbon materials [17,18,19,20], and natural polysaccharides [21,22]. Articles on the modification of natural polysaccharides, including chitin, chitosan, cellulose, alginate, mushroom polysaccharide, and starch, by liquid-phase plasma have been rapidly published in the past decade (Figure 1), which can imply the growth of the liquid-phase plasma in this field.

In this review, the insight into the plasmas in the liquid phase, mainly focused on an aqueous solution, is provided. The plasma technology called “solution plasma process (SPP)” will be dominantly explained. The discussion of recent development on the modification of chitin and chitosan, especially degradation, using the SPP will be given as examples. Modification techniques, reaction mechanisms, and changes in the properties of chitin and chitosan will be explained. In comparison with other existing methods, today, the modification of chitin and chitosan by the SPP is still at an early stage of development. The summary of relevant publications from the recent past to the present will provide benefits and a useful guideline for the researchers in the related fields to achieve the ecologically friendly and efficient method for the modification of chitin and chitosan. In addition, the remaining challenge and future trend of SPP technology in the field of not only chitin and chitosan but also other natural polysaccharides are also discussed to motivate future studies.

## 2. Solution Plasma Process (SPP): Chemistry and Influencing Parameters

**Figure 2:**
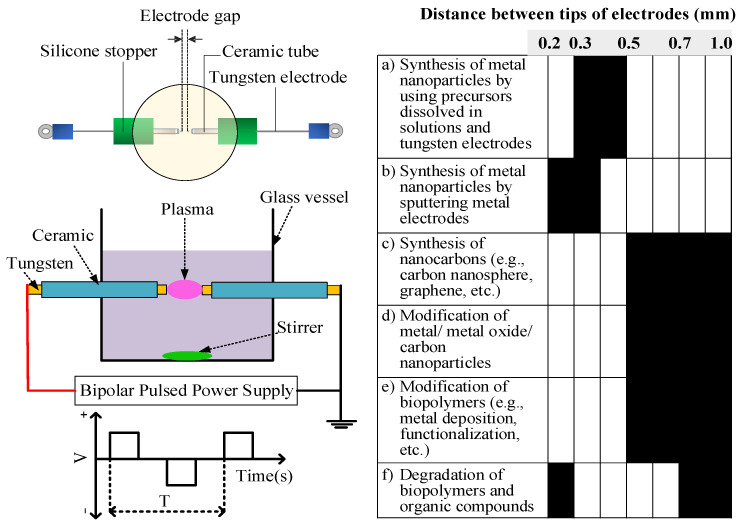
Schematic illustration of the SPP with pin-to-pin electrode configuration and its application with different distances between electrodes: (**a**) [14,29,30], (**b**) [31,32], (**c**) [27,33,34,35], (**d**) [36,37,38], (**e**) [15,22], and (**f**) [39,40,41,42].

Electrode geometric construction and phase patterns of liquid-phase plasmas can be categorized into four groups: (1) direct electrical discharge between two electrodes [23], (2) contact electrical discharge between electrodes and the surface of the surrounding electrolyte, (3) miscible electrical discharge with external gas injection, and (4) special excitation electrical discharge (i.e., radio frequency, microwave irradiation or laser ablation). In this section, we will discuss on the direct discharge between two electrodes under the liquid-phase, which are presented by various terms. For example, submerged liquid plasma (wire-to-plate configuration using direct current) [24,25], pulsed plasma in liquid (rod-to-rod configuration, using pulsed voltage) [23], and solution plasma (pin-to-pin and wire-to-plate configurations, bipolar pulsed voltage) [26,27]. Apparently, these terms are assigned following their experimental setup, electrical power source, and electrodes configuration. Henceforward, the solution plasma process (SPP) with pin-to-pin electrode configuration will be described. SPP with pin-to-pin electrode configuration was firstly proposed by Takai and Saito’s group [13,28]. In the SPP, the plasma is directly discharged between a pair of electrodes submerged under liquids at a short distance (0.2–1 mm), depending on its application, as shown in Figure 2. The power supplies are bipolar-pulsed high voltage supply. The use of pulsed voltage could reduce the current of the discharge. To a certain extent, it reduced the possibility of arc discharge due to thermal ion emission from the electrode and enhanced the number of carriers generated by secondary electron emission, thus enhancing the stability of discharge occurrence. Most studies using the SPP focus on the processing conditions and the properties and performance of the obtained products. The physical and chemical reactions occurring in the SPP are complex and rarely reported. Here, a brief explanation of the electrical discharge under an aqueous solution (e.g., electrical breakdown, formation of reactive species, and influencing factors) in the SPP is provided.

### 2.1. Electrical Breakdown

**Figure 3:**
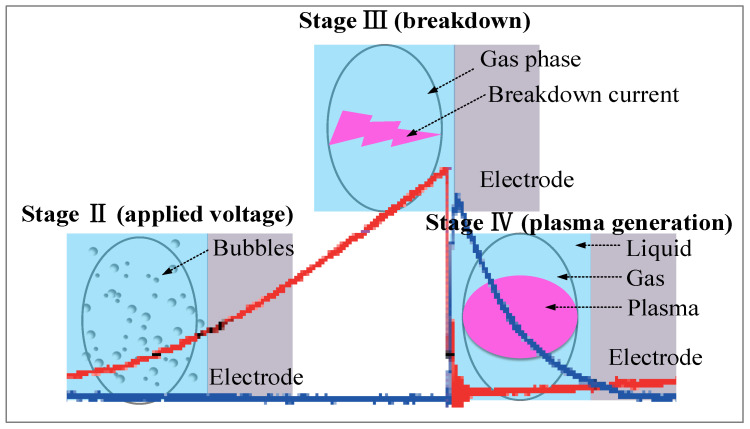
Schematic diagrams of the applied voltage, breakdown, and plasma generation stages in the SPP with the background of the current and voltage waveforms obtained by low-pass filter circuits [45].

In chemical physics, the difference between gas and liquid phases is the molecular density. The difference in molecular density causes different insulation to withstand capability, high collision frequency and energy dissipation rate, and low electron mobility in liquids. Therefore, the plasma chemistry in gas and liquid phases is significantly different. The physical mechanism of gas-phase plasma can be explained based on the electron avalanche [43], which is a process that free electrons in the medium are strongly accelerated by an electric field, resulting in the collision with other atoms or molecules and then ionization. However, the electron avalanche can rarely occur in the liquid mediums because liquids usually have high molecular density, low mobility of charges, and recombination rate. However, as mentioned above, the plasmas in the liquid phase can be carried. The explanation was previously clearly given by Saito et al. in 2008 [44]. The liquid (e.g., aqueous solutions) near the electrode tips turn to gas or the formation of bubbles due to Joule heating, which causes solution vapor and electrolysis, resulting in gases such as H_2_ and O_2_. When the bubble is formed, the electron avalanche is produced in bubbles. The electrical break down is developed inside the bubbles and then formed as the plasma channel, which is kind of like “unzipping”. The electrical breakdown can be controlled by the injection of more electrons into the ionization field in the bubble. Heo et al. revealed the current-voltage waveform by low-pass filter circuits, which could reduce noise signals as compared to that of the conventional circuits when the bipolar pulsed voltage was applied [45]. The stages of the applied voltages, breakdown, and plasma generation in the solution plasma process are also proposed in Figure 3.

Besides, the plasma discharges can be altered in different fluids to induce ionization, accelerate ions, and multiply the initial seed of electrons into the electron avalanche. The breakdown depends on the dipole moment of the fluids, including the dielectric behavior, ionization potential, band gap, and dipole moment [4,44]. For example, in water, the electrical discharge requires a large electric field (67–70 MV/m), while benzene requires a much higher energy field for the breakdown due to its higher dielectric strength (163 MV/m).

### 2.2. Formation of Reactive Species

**Figure 4:**
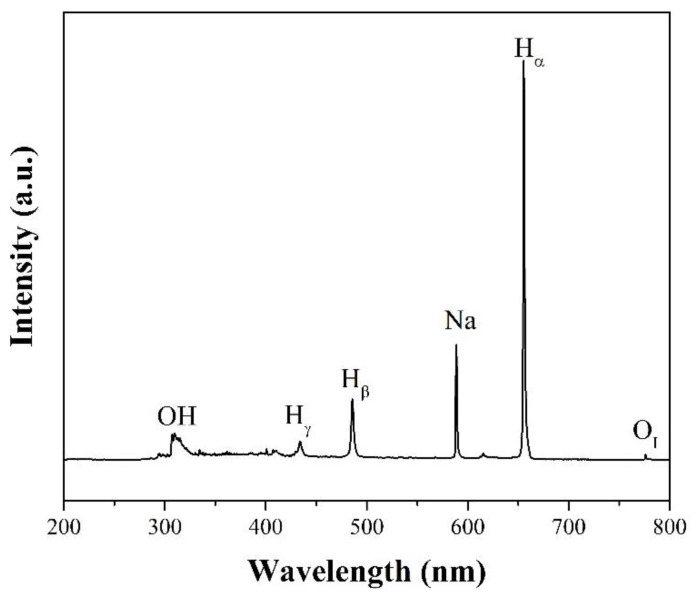
Optical emission spectrum (OES) of reactive species generated in water; its conductivity adjusted by NaCl at a concentration of 0.02 M during the SPP.

Considering how the SPP can be used to modify chitin and chitosan, the plasmas in aqueous solutions or water plays a key role because almost all modifying processes for chitin and chitosan have been reported in aqueous solutions [46,47,48,49]. Several reactive species are generated when the electrical discharge is carried in water through molecules collision, mass transfer, vaporization, sputtering, and ultra-violet (UV) [44]. Many previous studies demonstrated that both short-lived reactive species (e.g., hydroxyl radical (^•^OH) and hydrogen radical (^•^H), superoxide (O_2_^–^)) and long-lived reactive species (e.g., hydrogen peroxide (H_2_O_2_), ozone (O_3_)) are continuously formed and further reacted during the activation of water by the electrical discharge without the addition of a catalyst or chemical agents [50,51]. Figure 4 reveals the optical emission spectrum (OES) of reactive species generated in water during the SPP, which was measured in our research group. The short-lived reactive species, like ^•^OH and ^•^H, are firstly generated from the main reaction via electron impact dissociation and continuous collision of reactive species to surrounding molecules, as shown below in Equation (1) [52]. The activity of ^•^OH and ^•^H, is found to initiate and prolong modification reactions of chitin and chitosan, such as deacetylation, degradation, and altering the crystal structure, in not only plasma treatment but also in other methods, such as oxidative degradation [53]. Subsequently, hydrogen peroxide (H_2_O_2_), the most common long-lived reactive species in the plasma-activated water, is formed via the recombination of ^•^OH, as shown in Equation (12). In addition, in the water, there are dissolved oxygen molecules that can also be excited or ionized to reactive oxygen species (ROS), such as exited atomic oxygen (O(^1^D) or O_I_) and triplet ground-state atomic oxygen (O(^3^P)), singlet oxygen (^1^O_2_), and triplet oxygen (^3^O_2_). H_2_O_2_ and these ROS also evidently contribute to the formation of ^•^OH [51,52].

The reactions revealing the possible formation of ^•^OH, ^•^H, H_2_O_2_, and excited O are shown below [51,52]:

e^−^ + H_2_O → ^•^OH + ^•^H + e^−^ (Ionization of water molecule by plasma discharge) (1)

e^−^ + H_2_O → H_2_O* + e^−^ (Excitation of water molecule by plasma discharge) (2)

e^−^ + M → M*+ e^−^ (Sputtering of metal atom to excited metal atom) (3)

M* + H_2_O → ^•^OH + ^•^H + M (4)

e^−^ + O_2_ → O(^3^P) + O(^1^D) + e^−^ (Ionization of O_2_ molecule by plasma discharge) (5)

O(^1^D) + H_2_O → ^•^OH + ^•^OH (6)

^•^H + O_2_ → ^•^OH + O (7)

O + O → O_2_ (8)

UV + H_2_O → H_2_O* (Excitation of O_2_ molecule by UV) (9)

UV + H_2_O* → OH^–^ + H^+^ (10)

e^−^ + OH^–^ → ^•^OH + e^−^ (11)

^•^OH + ^•^OH → H_2_O_2_ (Recombination of ^•^OH to form H_2_O_2_) (12)

^•^OH + H_2_O_2_ → HO_2_ + H_2_O (13)

H_2_O_2_ → ^•^OH + ^•^OH (14)

### 2.3. Influencing Parameters in SPP under Aqueous Solutions

**Figure 5:**
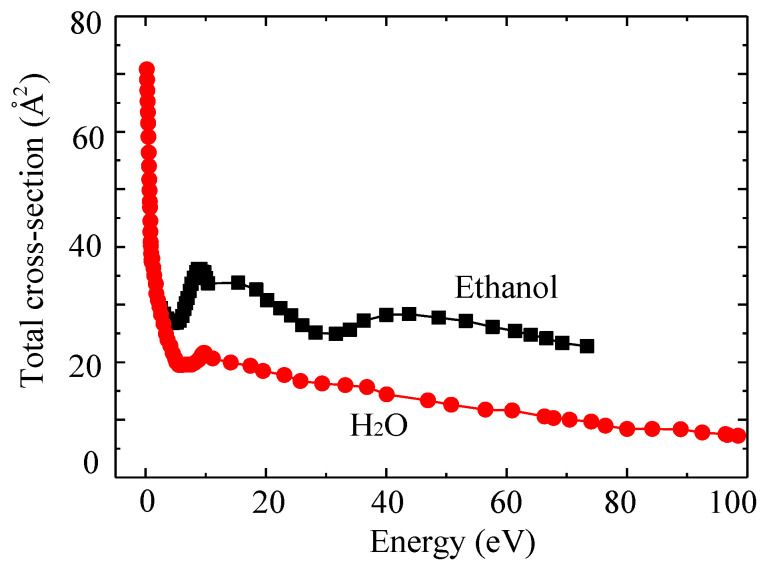
The total ionization cross-section of water and ethanol [60,61].

In the SPP, the electrical discharges under a different type of aqueous solutions, and by using different electrode types, and repetition frequency have been investigated and reveals the influence on the electrical breakdown and the formation of reactive species [44]. The number density of ^•^OH was measured in a bipolar electrical discharge between tips of wire-type tungsten electrodes in aqueous solutions containing HCl, KCl, and KOH at the same conductivity (500 μS/cm) by Miron et al. [54]. The breakdown voltage occurred at 920 V for HCl, 850 V for KCl, and 478 V for KOH, which implied that the energy required for the plasma breakdown was higher for the HCl solution than for the solution KCl and KOH. Additionally, the liquid conductivity measurement, which can suggest the ionization and dissociation of water to form hydrogen ions, was found to be relatively higher for the HCl solution. As the conductivity increased (up to approximately 520 μS/cm), the discharge became stronger, accelerating the erosion of the metal surface, due to the process of secondary release. The secondary release results in increasing the possibility of the collision from ions to metal electrode surface in the electron-rich sheath near the tip of electrodes and increases the collision possibility of ions [55,56]. This erosion causes an increase in the distance between the tips of electrodes. The distance between the tips of electrodes in the HCl solution increased from 0.1 mm to 0.17 mm, while 0.12 mm and 0.15 mm were observed in the KCl and KOH solution, respectively. Accordingly, it suggested that acidity has a significant influence on the sputtering of metal electrodes in the SPP, which is considered as an advantage for the synthesis of metal-based nanoparticles without using precursors and reducing agents [32,38,57]. Furthermore, the investigation on the formation of ^•^OH was also conducted via the time-resolved optical emission measurement at various time delays to the positive voltage pulse in the range of 0–38 μs. The density of ^•^OH generated in HCl solution was found to be high (2 × 10^17^ cm^–3^), while KOH, which is highly basic and can be an important source of ^•^OH, showed the lowest density (5 × 10^16^ cm^–3^). This may be caused by the collision of H^+^, which may easily move in the opposite direction of electron flow owing to low atomic weight, on the surface of metal electrodes, compared to that of K^+^. The collisions result in the sputtering and the release of electrons or excited metal atoms, which can further react with water molecules and promote the generation of ^•^OH via the reactions showed above in Equation (4). In the nanosecond pulsed plasma discharge system for water-film plasma reactor, the influence of liquid conductivity on the electrical breakdown and the production of H_2_O_2_ was also studied by Wang et al. [58]. The breakdown voltage was found to be decreased with increasing liquid conductivity (from 100 μS/cm to 36,000 μS/cm). This was explained by the decrease of dielectric relaxation time, which is the time scale of relaxation for moving charge carriers in materials. Increasing the liquid conductivity reduced the resistance of the liquid and facilitated the current to flow through the liquid when the electrical field was applied at the electrodes. This also caused the increment of energy dissipation into the bulk liquid. However, the production rate of H_2_O_2_, which can be a source for ^•^OH, did not change significantly with conductivity. Moreover, the mixture of aqueous solution and organic solvent, like ethanol, was also investigated by Takeuchi et al. [59]. The breakdown was delayed, and the time needed until the steady-state discharge (constant current) was longer for the mixture with 50% ethanol, compared to the aqueous solution alone. This is because the ionization energy of an ethanol molecule (16 eV) is higher than that of a water molecule (13 eV). The total ionization cross-section of ethanol is larger than that of water with electron energy higher than 16 eV [60,61], as shown in Figure 5.

However, the discharge probability increased with the addition of ethanol to the solution and the power required to produce bubbles and initiated a discharge that decreased with the addition of ethanol. This could be caused by the lower boiling temperature of ethanol, as compared to that of water. Furthermore, the discharge in the solution with ethanol (50%) was found to produce smaller-size bubbles than that obtained in the solution without ethanol, which resulted in easier sustaining discharges and higher discharge probability higher. Besides, the OES spectra from solution without ethanol and with ethanol showed ^•^OH, ^•^H, and ^•^O, while the solution with ethanol showed relatively weak signals of ^•^OH and ^•^O. However, the emissions of C_2_ swan band (440 nm) and ^•^CH (431 nm) could be observed in the solution with ethanol, due to the dissociation of ethanol vapor. This evidence shows benefit in the synthesis of carbon nanomaterials via the solution plasma process, similar to the systems with other organic solvents [27,62]. According to the evidence previously showed, it pointed out that the choice of solutions, which include conductivity, pH, and type of ions, exhibits a significant effect on the properties of plasmas in liquids.

**Figure 6:**
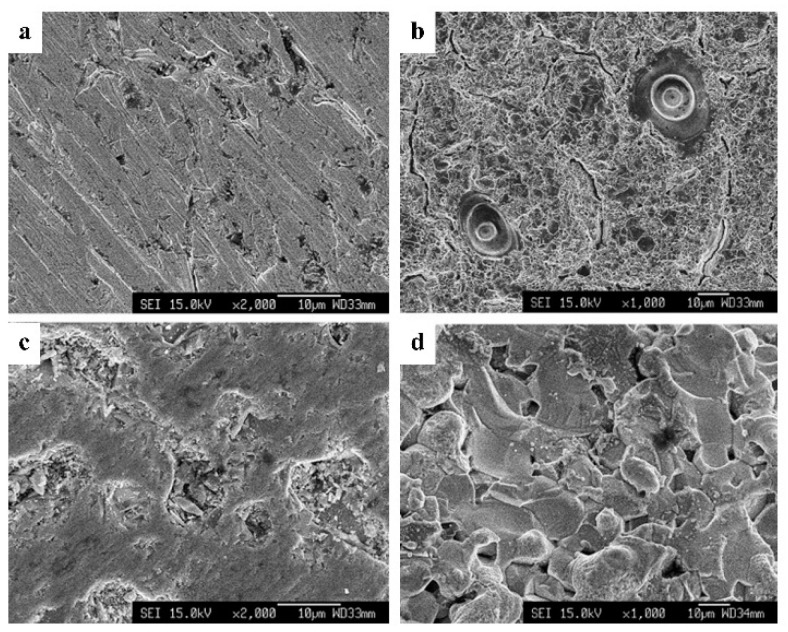
SEM images of the surface of the W electrodes (**a**) before and (**b**) after being exposed to 10 min of the discharge and SEM images of the surface of the LaB_6_ electrodes (**c**) before and (**d**) after being exposed to 10 min of the discharge [64].

Types of electrodes also showed a significant effect on the electrical breakdown and generation of ^•^OH in the solution plasma process. In 2010, Miron et al. studied the current-voltage characteristics and the optical emission spectroscopy in the solution plasma process by using tantalum (Ta) and tungsten (W) electrodes with the circulated and non-circulated water systems [63]. In comparison, the energy required for the electrical breakdown was also found to be higher for the systems with Ta electrodes. The current-voltage characteristics for all systems showed the features of a spark discharge, and the transition to arc plasma was observed in the case of using Ta electrodes. Furthermore, according to the OES spectra, the transition of the ^•^OH was found to be different for the circulated water and the non-circulated water discharges when different electrodes were used in the discharge. For W electrodes, the band of the ^•^OH was detected to be the strongest at 307.8 nm, while the band was strongest at 312.6 nm for the Ta electrodes. The reason for this phenomenon was explained relating to the different plasma temperatures in the case of W and Ta electrodes. Moreover, the board emission continuum spectra in the range spectra in the range of 350–940 nm were exhibited only for Ta electrodes, which might be due to the eroded and heated metal particles from the metal surface at the tips of electrodes. Later, in 2011, Miron et al. also studied the influence of electrodes made of W and lanthanum hexaboride (LaB_6_), on the electrical breakdown in water [64]. The polished asperities of electrodes showed different morphologies, which caused the difference of electric field required for the electrical breakdown (~190 kV/cm for W electrodes and ~160 kV/cm for LaB_6_). The rougher surface of LaB_6_ shown in Figure 6 had a higher number of locally concentrated emission sites than that of W electrodes. In addition, the melting points of these two materials (3422 °C and 2220 °C for W and LaB_6_, respectively) were believed to be the factor for the erosion of electrodes, which resulted in the formation of different reactive species under corresponding plasma condition. Even though the erosion of metal electrodes could cause contamination to final products in the modification process by the SPP, this phenomenon has also given benefits to the synthesis of metal nanoparticles without using precursors and reducing agents [32]. The influence of electrode types on the generation of reactive species has been continuously investigated. Goto et al. also investigated the electrical discharge in water by using copper (Cu) electrodes. They found the emission line of atomic Cu in the optical emission spectra and proposed that the Cu atoms may act as a catalyst to promote the generation of ^•^OH [51]. Besides, not only the influence of electrode types but also the polishing of electrodes is also an essential step for the SPP. Yui et al. reported that the sharpening could increase the stability of the spatial position of the plasma [5]. Accordingly, the management of electrode tips, such as polishing surface and sharpening, should be done, depending on the application. Sharpening electrodes that can provide the high stability of plasma may be suitably applied in precisely controlled chemical reactions. Meanwhile, reactions like degradation require a large plasma with high production of reactive species, which polishing surface of electrode tips suffices.

The plasma parameters, such as frequency, were found to be related to the relative amount of injected energy per pulse [20]. Unfortunately, the investigation was conducted by applying the pulse frequency using the bipolar power supply (from 25 to 65 kHz) in only benzene, which led to the formation of carbon materials. The result showed that there were two different operation regimes. The first regime, characterized as glow discharge, occurred when the pulse frequency ranged between 25 and 50 kHz was applied, which referred to lower energy input at a certain interval. In comparison, the second regime, characterized as spark discharge, could be obtained by applying the pulse frequency of 65 kHz, which could result in relatively high energy input in the corresponding period. The plasma/gas temperature was reported to be increased with increasing energy input, which might influence the obtained carbon products from each regime to reveal different morphology. Therefore, it is possible that the electrical discharge in aqueous solutions can also be influenced by applying different frequencies.

**Figure 7:**
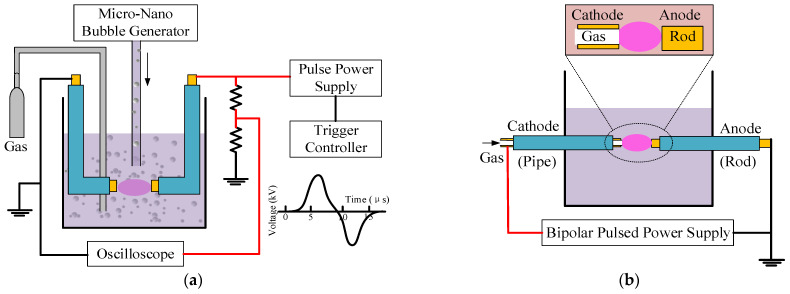
Systems with different configurations of adding bubbles, proposed by (**a**) Goto et al. [51] and (**b**) Yui et al. [65].

**Figure 8:**
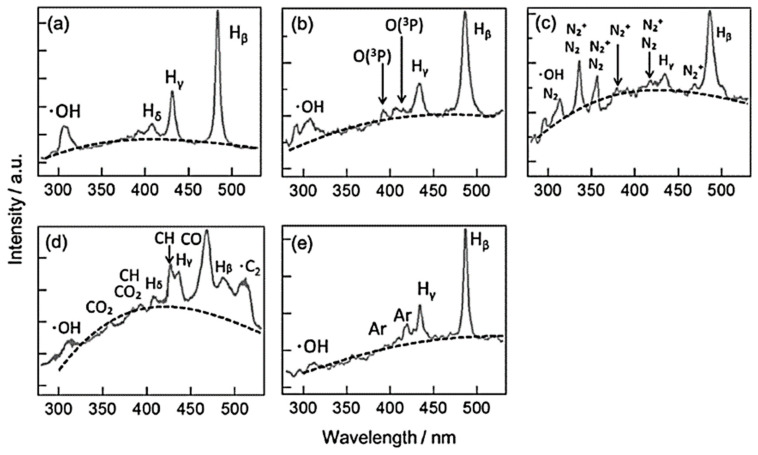
OES spectra from the solution plasma process without the gas injection (**a**), and with the injections of O_2_ (**b**), N_2_ (**c**), CO_2_ (**d**), and Ar (**e**). The spectra were obtained with an integration time of 12.8 s, and 320,000 pulses were integrated during the time span. The black dotted lines represent the best-fitted curves with the blackbody radiation [65].

Many researchers also attempted to enhance the efficiency of electrical discharge under aqueous solutions by adding bubbles into the system with different configurations, as revealed in Figure 7a,b. The configuration in Figure 7a was proposed by Goto et al. They studied the formation of hydrogen peroxide, which is a powerful oxidant, by applying bipolar pulsed voltage with fine O_2_ bubbles [51]. The result showed that the high concentration of dissolved O_2_ insignificantly increases the amount of H_2_O_2_. Moreover, Yui et al. developed a direct gas injection system at the tip of the electrode, as shown in Figure 7b [5,65]. The injecting gases were O_2_, CO_2_, N_2_, and Ar. The injection of gases caused the spatial fluctuation of plasma. The electron number density in the plasma was found to be increased by injecting O_2_, CO_2_, and N_2_. It was because the generation of electrons in the plasma was increased by the enhanced collision between the positively charged ions, which can be accelerated by the applied voltage, and other particles, ions, and the cathodic electrode. The kinetic impulses of the collision increased due to the injected gases with larger molecular weights than H_2_O. On the other hand, the injection of Ar gas resulted in the reduction of electron number density. This is because Ar is an inert gas that has a larger ionization energy of 1500 kJ/mol, as compared to other gases, such as O_2_ (1175 kJ/mol). The contents of the plasma, characterized by the OES, were found to be different, as illustrated in Figure 8.

## 3. Solution Plasma Process for Modification of Chitin and Chitosan

### 3.1. Chitin and Chitosan

**Figure 9:**
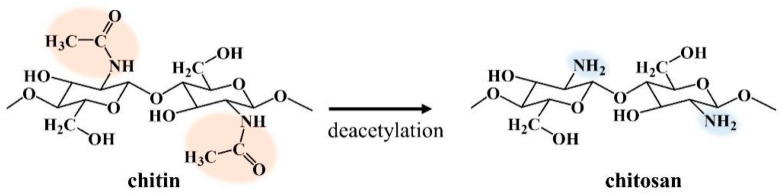
Chemical structures of chitin and chitosan.

**Figure 10:**
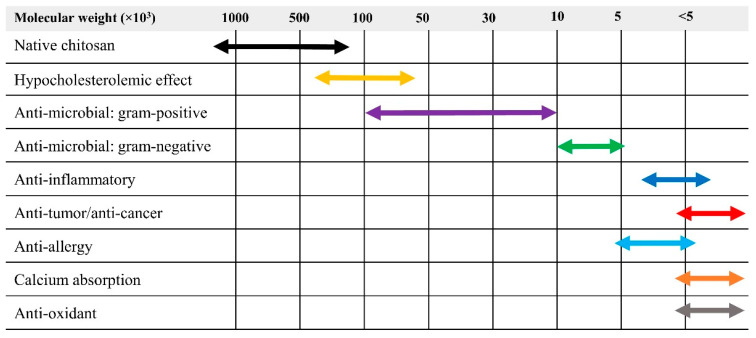
Biological properties of chitosan, depending on its molecular weight. (Note: native chitosan [39,41,67,74], hypocholesterolemic effect [75], anti-microbial: gram-positive [76], anti-microbial: gram-negative [76], anti-inflammatory [77], anti-tumor/anti-cancer [39,49,74,78], anti-allergy [79], calcium absorption [80], anti-oxidant [81]).

Chitin was first discovered in the early nineteenth century. It can be extracted from crustacean shell waste (e.g., shrimp and crab shells), insects, and plants [66,67]. Similar to cellulose, it functions as a structural linear polysaccharide. Unlike cellulose, it contains acetamido and amino groups at C–2 position on its pyranose rings [68]. Figure 9 shows the chemical structure of chitin consisting of 2-acetamido-2-deoxy-β-D-glucopyranose as a major repeating unit and glucosamine connected by β (1→4) linkages. Chitosan is one of the most studied chitin derivatives, which is obtained by deacetylation of chitin [69]. Due to the deacetylation reaction, acetyl groups are removed and converted to amino groups, as revealed in Figure 9. The presence of amino groups in the chitosan structure is responsible for its unique functional and biological properties, depending on its molecular weight, as revealed in Figure 10 [70,71,72]. Moreover, chitin and chitosan also have interesting characteristics, such as biocompatibility, non-toxicity, low allergenicity, and biodegradability. However, their original chemical structures (e.g., high molecular weight and strong hydrogen-band network) cause poor solubility in organic solvents and water, which limit utilization in several fields, especially in biomedical applications [73]. To improve their properties and broaden their applications, chitin and chitosan are intensively studied and modified. For example, degradation of chitin and chitosan to obtain water-soluble degraded products (e.g., chitooligosaccharides), and chemical modification of its functional groups (e.g., deacetylation, carboxymethylation).

Several protocols can be used to modify chitin and chitosan. In general, they can be categorized into three main methods: (i) chemical method [82,83], (ii) enzymatic method [84,85], and (iii) physical method [86,87,88]. Chemical methods typically give high efficiency or high rate in the modification of chitin and chitosan. However, their drawbacks are concerned with the cost of chemicals, waste management, and severe reactions, resulting in unwanted products (e.g., some over-degraded products). Enzymatic methods can provide a mild reaction and selectively modify chitin and chitosan [89]; for example, it can produce specific oligomers of chitin and chitosan, as shown in Table 1. However, the process is time-consuming, and the cost of handling is relatively high. In recent years, exploring alternative techniques for chitin and chitosan modification, physical methods for the modification of chitin and chitosan, have focused on the utilization of various kinds of energy, including irradiation [90], sonication [91], microwave [92], and plasma. These methods can provide rapid reactions with relatively lower chemical uses and low contamination in the final product, compared to enzymatic and chemical methods, respectively. Among these methods, liquid-phase plasma treatment of chitosan in aqueous solutions is believed to be a novel and effective method, examples of which are given in Table 1 and Table 2 (in case of degradation of chitosan). Therefore, it should be clearly understood, aiming at further development.

### 3.2. Reduction of Molecular Weight and Destruction of Crystallinity Via SPP

There are several SPP parameters that have been investigated to understand the degradation process to reduce the molecular weight of chitosan, such as reaction time, electrode configuration, types of electrodes, and frequency. In 2012, the SPP was introduced for the first time to reduce the molecular weight of chitosan by Prasertsung et al. [46]. Chitosan is insoluble in water and organic solvents, but it is soluble in dilute aqueous acidic solution at pH < 6.5. The dissolution resulted in the protonation of the amino group (R–NH_2_) in glucosamine units into soluble form R–NH_3_^+^. To obtain a homogeneous reaction solution, chitosan was dissolved in acetic acid prior to the SPP. The molecular weight of chitosan sharply dropped in the beginning (0–60 min) and then gradually decreased, approaching a constant. Reactive species, like ^•^OH, was believed to play an important role in the molecular weight reduction of chitosan. As a result, the longer time of the reaction had a lesser effect on the reduction of molecular weight because the number of short-chain chitosan increased while the number of ^•^OH produced in the system remained the same according to the fixed SPP condition. Moreover, they also reported that the main structure (pyranose ring) was not altered after the solution plasma process. Besides, Prasertsung et al. also applied the SPP on the molecular weight reduction of β-chitosan [40]. β-chitosan has a low hydrogen-bonding network, leading to loss in crystalline structure, compared to normal chitosan, which mostly refers to β-chitosan. Compared to their previous study using α-chitosan, the reaction rate was higher. The molecular weight of β-chitosan (5.5 × 10^5^) was markedly decreased from almost 4 times (1.5 × 10^5^) and 30 times (1.9 × 10^4^) after the solution plasma treatment for 30 min and 300 min, respectively. The water solubility of the obtained products was also found to be improved. Accordingly, the tuning of the SPP treatment time showed potential to obtain chitosan with specific molecular weight, which can be further used in various applications, as shown in Figure 10.

**Figure 11:**
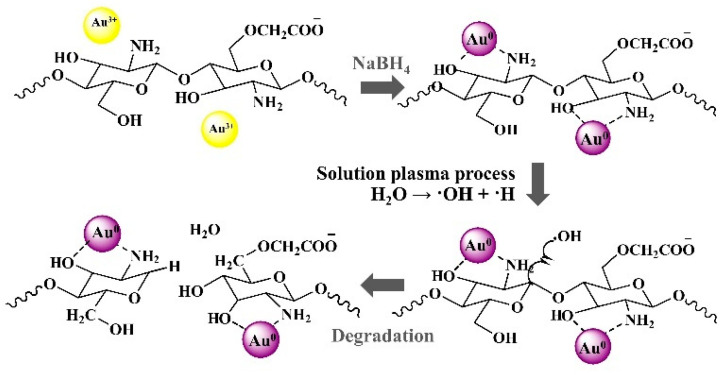
Illustration of a formation of N,O-carboxmethyl chitosan decorated with gold nanoparticles and a possible degradation mechanism by hydroxyl radicals generated by the SPP [49].

**Figure 12:**
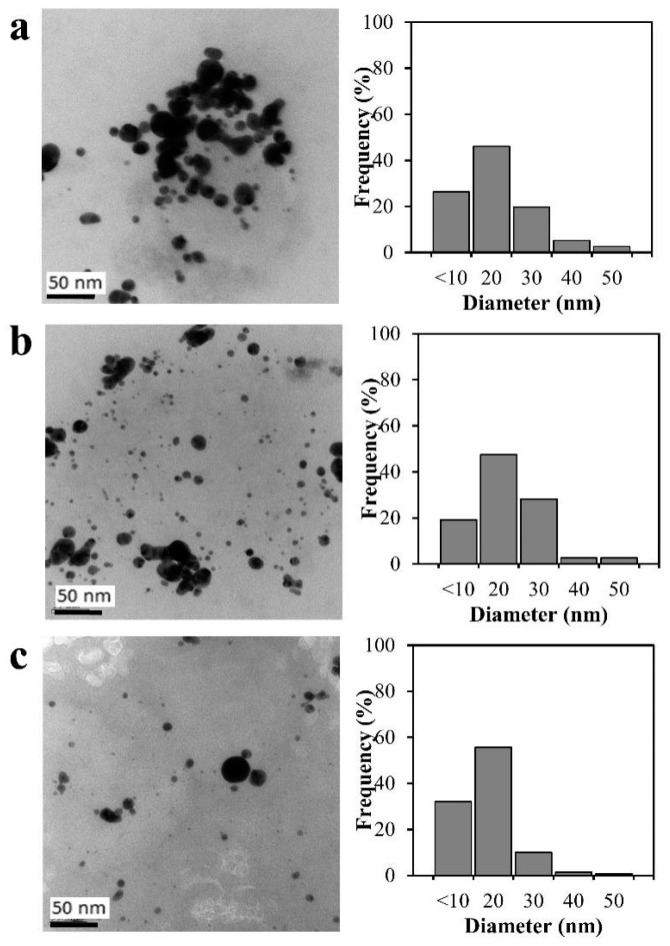
TEM images of the N,O-carboxmethyl chitosan decorated with gold nanoparticles (**a**) and the degraded products after degradation by the SPP for 45 (**b**) and 90 min (**c**) [49].

In addition, the effect of SPP on the chitosan derivatives, N,O-carboxymethyl chitosan decorated with gold nanoparticles, water-soluble chitosan with a highly negative charge, was also studied by Chokradjaroen et al. [49]. In their study, chemical reduction of Au^3+^ was firstly conducted in N,O-carboxymethyl chitosan solution, as shown in Figure 11. The aggregation of gold nanoparticles could be observed. However, after the SPP, the distribution of gold nanoparticles was improved and their average size was also reduced from 11 nm to 9 nm, as shown in TEM images (Figure 12). A similar result was also reported and explained that as the SPP prolonged, the pH of the solution decreased, leading to the partial dissolution of metal nanoparticles [28]. Not only the change in the size of gold nanoparticles but also the hydrodynamic size, which referred to the molecular weight of N,O-carboxymethyl chitosan, was also influenced by the ^•^OH formed in the system. The chain scission of N,O-carboxymethyl chitosan was occurred by the attacking of ^•^OH to C-1 position of chitosan, resulting in the bond breakage at β-glycosidic linkages, as shown in Figure 11. After the reaction, the result of chemical structure analysis showed that there was no destruction of the main structure and functional groups, including carboxymethyl group and interaction with gold nanoparticles. Nevertheless, the negative charge of N,O-carboxymethyl chitosan decorated with gold nanoparticles was lowered in magnitude, according to the Zeta potential measurement. Consequently, the obtained products were evaluated and showed enhanced cytotoxicity and improved the selectivity toward cancer cells than normal cells.

In addition to the effect of reaction time, the effects of SPP conditions (e.g., electrode types and pulse frequency) also play an important role in the degradation rate and properties of chitosan. Prasertsung et al. used various types of electrodes, including tungsten (W), copper (Cu), and iron (Fe), and varied the applied pulse frequency of the bipolar supply from 15 to 30 kHz [47]. According to the obtained result, the different electrode types differently affected the degradation of chitosan. Within 60 min, the molecular weight of chitosan could be reduced from 1.3 × 10^5^ to 8.7 × 10^4^, 6.2 × 10^4^, and 4.1 × 10^4^ for the system with W, Cu, and Fe at 15 kHz, respectively. The melting points of W, Cu, and Fe are 3422 °C, 1084 °C, and 1204 °C. A stronger promoting effect of the system with Cu and Fe electrodes could be attributed to the metal atoms or ions from the erosion of the metal surface at the tip of electrodes during plasma treatment. Especially, metal atoms and ions from iron electrodes could be transformed into ferrous ions and effectively participate in the Fenton reaction. The decomposition of H_2_O_2_ generated in the system led to the increment of ^•^OH. Besides, the pulse frequency was found to significantly influence the reduction of the molecular weight of chitosan. After the solution plasma process at the applied pulse frequencies of 15, 22.5, and 30 kHz, the molecular weights of the obtained products were 1.3 × 10^4^, 9.2 × 10^3^, and 6.8 × 10^3^, respectively. This could be described by the raising of the energy input when the pulse frequency increased. However, the molecular weight distributions of the obtained products were still relatively high, which is not suitable for many applications. The polydispersity index (PDI) ranged from 2.5 to 3.5, which was higher than the ideal PDI (1). In biomedical applications, monodisperse low molecular weight chitosan or chitooligosaccharides, COS, (PDI = 1) is desired.

**Figure 13:**
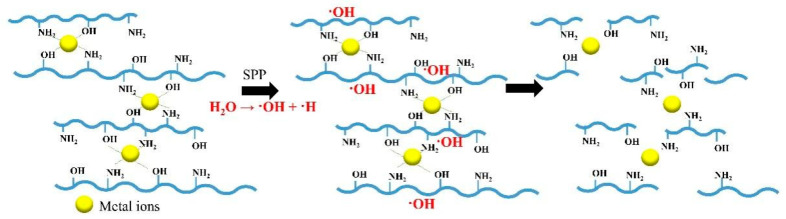
Degradation of chitosan–metal complexes via SPP.

To acquire the specific product of low-molecular-weight chitosan with high purification, several techniques were studied in combination with the SPP. For example, Pornsunthornthawee et al. used the benefit of the chitosan-metal complex to induce the selective chain scission. Metal ions, such as silver ion (Ag^+^), zinc ion (Zn^2+^), copper (II) ion (Cu^2+^), and ferric ion (Fe^3+^), were used to form complexes with chitosan, which was dissolved in the acid solution, at a metal-to-chitosan molar ratio of 1:8 [41]. The hydroxyl groups (‒OH) and amine (–NH_2_) groups in the chitosan structure can act as good ligands for coordination with the metal ions. This coordination usually causes the weakening of covalent bonds near the coordinating site, leading to weak points which can promote the chain-scission reaction, as shown in Figure 13. As a result, the complexation of chitosan with Cu^2+^ or Fe^3+^ ions strongly promoted the degradation rate of chitosan, while chitosan–Ag^+^ and chitosan–Zn^2+^ complexes exhibited slight change, compared to chitosan alone. After the SPP treatment for 180 min, the only complexation with either Cu^2+^ could produce glucosamine and COS with a molecular weight of 10^3^ and PDI of 1.4. However, the reaction time was quite long, and the separation of the metal ions from the glucosamine and COS was required, prior to further use.

Later, the SPP was also combined with an environmental-friendly oxidizing agent (i.e., H_2_O_2_) and O_2_ bubbling in order to enhance the rate of reaction and lower the possibility of contamination to the final products, as well as move toward a greener process. Chokradjaroen et. al. found that the combination of SPP and 4 mM H_2_O_2_ could effectively promote the chain scission of chitosan, resulting in the significant decrease of molecular weight (from 450 × 10^3^ to 50 × 10^3^) within 60 min [74]. The degradation mechanism of chitosan by applying the SPP in combination with H_2_O_2_ was also proposed in this work. They explained that excitation and ionization of H_2_O molecules should mainly occur during the plasma discharge since the major component in the system was H_2_O molecules. Electrons emitted from ionization continuously collided with the surrounding H_2_O molecules to produce ^•^OH. The addition of H_2_O_2_ could promote the reaction because H_2_O_2_ itself can dissociate to form ^•^OH. This phenomenon helps to increase the ^•^OH concentration in the system and enhance the degradation of chitosan. Due to the relatively short reaction time and not too severe reaction, the prevention of over-degradation could be done. The obtained COS has an average molecular weight of 1.44 × 10^3^ (8 oligomers), which has been reported to be suitable for anticancer activity. Moreover, Ma et al. reported on the effect of bubbling gas added in the solution plasma process on the molecular weight and physicochemical properties of chitosan [107]. They found that when the bubbling gas (i.e., O_2_) was presented in the SPP system, the concentration of ^•^OH increased, which caused not only the enhancement of molecular weight reduction but also influence on physical properties (e.g., destruction of crystallinity). Moreover, the SPP combined with H_2_O_2_ and O_2_ did not show a significant change on the pyranose ring and functional groups (–OH, –NH_2_, etc.) of chitosan, which is a key possessing the biological properties.

Several methods (e.g., microwave, sonication, and irradiation) have been claimed as green degradation of chitosan. However, in these methods as well as most studies using the SPP, chitosan is mostly dissolved in an acetic acid solution (approximately 1–2 M), to obtain a homogeneous chitosan solution prior to the degradation, as shown in Table 2. After the degradation, separating water-soluble COS from the high-molecular-weight chitosan is usually found to be complicated because they are dissolved together in the solution. Chokradjaroen et al. realized this issue; therefore, they had tried to propose a technique that can effectively produce chitosan with specific molecular weight, reduce the chemical use in the system, and simplify the post-treatment step, including the separation and purification processes. Accordingly, the heterogeneous degradation of chitosan by the SPP was proposed. For example, the heterogeneous degradation of chitosan hydrogel, which could hold a large amount of water in its three-dimensional networks, was conducted by the SPP with the presence of carboxylic acids (i.e., monocarboxylic acid, dicarboxylic acid, and tricarboxylic acid) at an acid-to-chitosan mole ratio of 1-to-8 (~1.55 mM) [106]. The chitosan hydrogel with addition of carboxylic acids was found to have good mobility in the SPP reactor. According to the molecular weight of the obtained products, the number of carboxylic groups in the carboxylic acid exhibited the effect on the rate of reaction, molecular weight, and PDI of the obtained COS. Acetate anions (CH_3_COO^−^) are small, which should be able to penetrate the three-dimensional networks of chitosan hydrogel and undergo ionic interaction with the protonated amino group (–NH_3_^+^), leading to electrostatic repulsion and expansion between chitosan chains. The expansion was believed to facilitate the accessibility of ^•^OH to C-1 position of chitosan. Besides, all dicarboxylic acids and tricarboxylic acid can dissociate in the water, based on their pKa. Therefore, ionic interactions between the –NH_3_^+^ groups of chitosan and the –COO^−^ groups of the carboxylic acids can occur. This can lead to ionic crosslinking or complexation of dicarboxylic and tricarboxylic acids with –NH_3_^+^ groups of some adjacent chitosan chains, which should result in the weakening of the covalent bonds near the complexed sites. The COS obtained from the system with dicarboxylic acid had a similar molecular weight of approximately 2100 (PDI = 1.8), while the tricarboxylic acid system could produce COS with 1500 (PDI = 1.5). As a result, the complexation of dicarboxylic and tricarboxylic acids showed potential for the selective degradation of chitosan. Moreover, due to the use of carboxylic acids with an incredibly low concentration in the reaction, further purification after the centrifugation was unnecessary. The overall process became much simpler, compared to that of the homogenous chitosan solution, like in other studies. In addition, carboxylic acids were reported to be safe for use in food and drug production [108].

**Figure 14:**
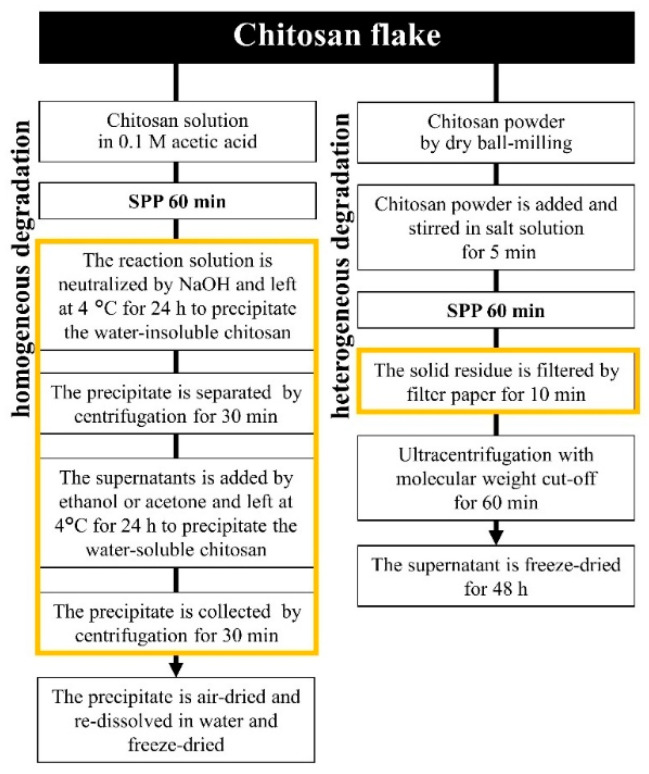
Flow chart of comparison between homogeneous and heterogeneous degradation of chitosan by the SPP [39,74].

**Figure 15:**
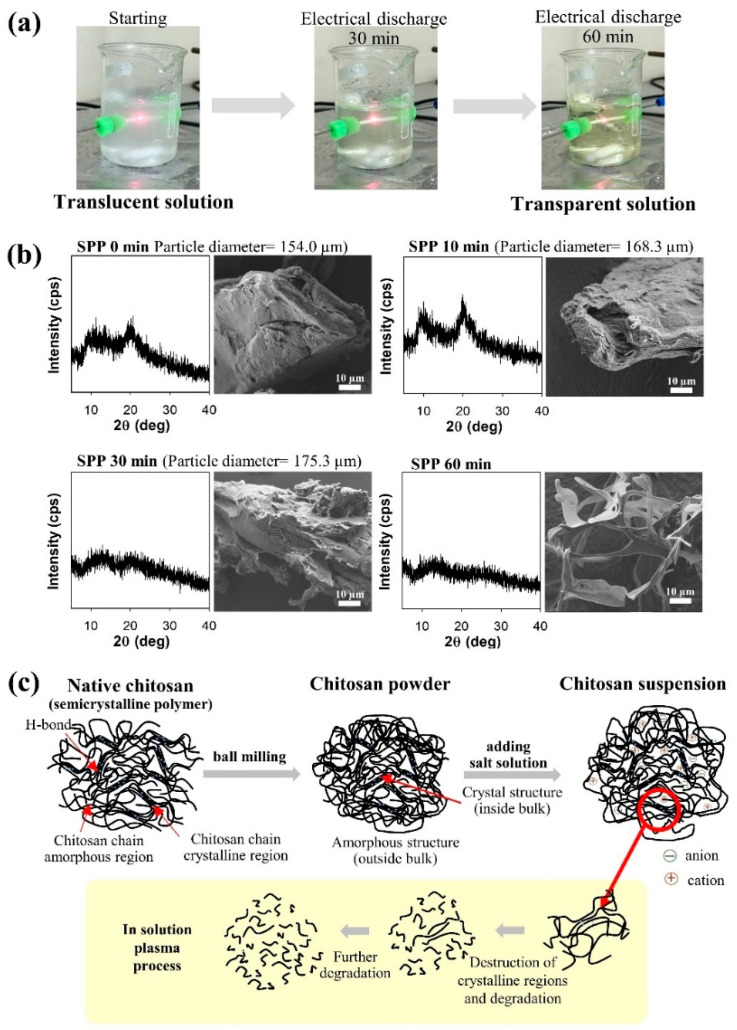
(**a**) Digital images of chitosan powder degradation by the SPP, (**b**) XRD and SEM results of the obtained products from chitosan powder degradation by the solution plasma process, and (**c**) Proposed destruction and degradation mechanism [39].

Furthermore, the fine power of chitosan was used and provided a good dispersion in the SPP reactor and led to the simpler degradation process of chitosan, compared to other previous techniques, as shown in Figure 14 [39]. A variety of sodium salts (e.g., NaCl, NaI, NaNO_3_, and Na_2_SO_4_) and metal chloride (e.g., CaCl_2_, MnCl_2_, and CeCl_3_) used in this work exhibited the different influence on the rate of degradation on the main structure (i.e., pyranose rings and functional groups) of chitosan. According to the result, the inorganic salts, such as Na_2_SO_4_, NaCl, and NaNO_3_, could promote stable plasma formation as well as the molecular weight reduction of chitosan. After the plasma discharge, water-soluble and water-insoluble products can be easily observed, as revealed in Figure 15a. The morphology and crystallinity of the plasma-treated chitosan were also observed as a function of time (Figure 15b). The result showed evidence that both the degradation and destruction of crystallinity occurred simultaneously, as proposed in Figure 15c. For the presence of NaI, MnCl_2_, and CeCl_3_ in the reaction solutions, they could not provide the effective molecular weight reduction of chitosan powder. In general, NaI can be dissociated to iodide ion (I^–^), which can be oxidized to form iodine molecules. Meanwhile, MnCl_2_ and CeCl_3_ are a transition metal and a lanthanide, respectively, which have several oxidation states. Therefore, they can undergo some redox reactions. These reactions could probably compete with the degradation reaction, leading to the lowering of the degradation efficiency. The obtained COS products in their works were analyzed and evaluated for their cytotoxicity and showed that they were highly purified and had potential as a nature-derived anticancer agent.

Recently, the role of reactive species generated in the SPP system was intensively investigated by Ma et al. by using a technique based on radical scavenging (radical ^•^OH scavenger, tert-butanol; H_2_O_2_ scavenger, MnO_2_; radical ^•^O scavenger, 1,4-benzoquinone; hydrated electrons scavenger, NaH_2_PO_4_) [109]. The result showed that not only ^•^OH but also ^•^O and H_2_O_2_ participate in the degradation of chitosan, while the hydrated electron played a partial role. The addition of ^•^O and H_2_O_2_ scavengers were found to significantly inhibit the degradation of chitosan, compared to that of ^•^OH scavenging. This might be because when ^•^O and H_2_O_2_ were scavenged, it might enhance consumption and lower the production of ^•^OH, respectively.

### 3.3. Deacetylation of Chitin Via SPP

**Figure 16:**
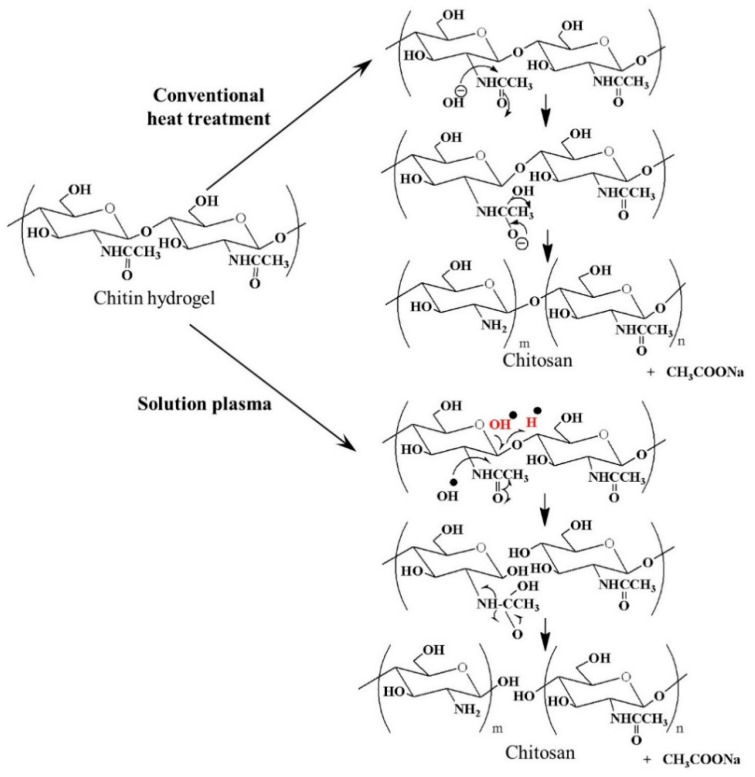
Comparison between deacetylations by conventional heating with NaOH (40–50%) and SPP using NaOH (1–12%) [112].

Deacetylation is considered as a first step to functionalize chitin, which normally cannot dissolve in water and organic solvents, into other various derivates (e.g., chitosan). For several decades, the deacetylation of chitin converting an acetamido group at the C–2 position of a pyranose ring to an amino group has widely been conducted by conventional heat treatment (100–160 °C) using 40–50% NaOH, especially in commercial scale. For example, Kurita et al. used 50% NaOH solution at 130 °C [110], and Galed et al. used the corresponding concentration of NaOH at 110 °C to convert chitin to chitosan [111]. Recently, the SPP was introduced and could provide effective deacetylation of chitin with much lower concentration of chemicals (i.e., 1–12% NaOH) [112]. The key for this green process was the plasma-generated reactive species, including ^•^OH and ^•^H. The comparison of deacetylation via conventional heat treatment and SPP is revealed in Figure 16. Chitin hydrogel was used as a starting material and dispersed in NaOH/methanol/water solution (i.e., 90% methanol/water solution containing 12% NaOH). During the plasma discharge, ^•^OH and ^•^H were proposed to be generated via the following reactions:

For the conventional heat treatment,

NaOH → Na^+^ + OH^−^ (15)

For the SPP,

H_2_O → ^•^OH + ^•^H (16)

ROH → ^•^R + ^•^OH (17)

After the plasma treatment for 5 h, it was found that the degree of deacetylation changed from 35% to 78%, and the molecular weight of chitin decreased from approximately 10^6^ to 2 × 10^5^, leading to the improved solubility in diluted acetic acid (2%) and possessing antibacterial activity. Although the degree of deacetylation of the products was still not competitive with that obtained from the conventional heat treatment with a high concentration of NaOH, the finding in this study opened an opportunity for the further development of a more environmentally friendly process for the industrial-scale production of chitosan.

## 4. Conclusions and Future Aspects

In this paper, the fundamental electrical discharge in water and aqueous solutions using pin-to-pin electrode configuration, referred to as the solution plasma process (SPP), is discussed. A summary of parameters influencing the electrical breakdown, plasma stability, and reactive species formation is also given. The given fundamental is hoped to be used as a guideline for designing experimental setups and procedures for the SPP, aiming to obtain an effective process not only for modification of chitin and chitosan but also other kinds of application. Moreover, various strategies for the modification of chitin and chitosan, shown in this paper, update the development progress, as well as give ideas for further development to change low-value materials to high-value materials. Even though the production of high-purified chitooligosaccharides via the SPP has been accomplished, selective chain scission of chitosan and chitin to produce chitooligosaccharides with a narrow polydispersity index is challenging. Moreover, the modification of chitin and chitosan via the SPP is still in a beginning stage. It is believed that there is plenty of room to apply the SPP to modify functional groups or pyranose rings of chitin and chitosan, including deacetylation carboxylation, sulfonation, etc.

## Figures and Tables

**Table 1:** Degradation of chitin and chitosan via chemical and enzymatic methods.

Chemicals	Chemical Concentration	Temperature	Time	MW/ Products	Ref.
Hydrochloric acid	35%	80 °C	1.4 h	3–5 × 10^3^	[93]
Hydrochloric acid	12 M	40 °C	8 h	1–3 × 10^3^	[94]
Nitrous acid	70 mM	0 °C	9 h	2–6 × 10^3^	[95]
Hydrogen fluoride	100%	20 °C	19h	~2 × 10^3^	[96]
Hydrogen peroxide	30%	70 °C	2 h	1.7–3.81 × 10^3^	[97]
Chitosanase (Bacillus pumilus BN-262)	0.1 M NaOAc buffer, pH 5.3	37 °C	96 h	2–3 oligomers	[98]
Chitinase (Vibrio furnissii)	Chitin in DMSO/LiCl, pH 7.9	37 °C	24 h	2 oligomers	[99]
Papain	NaOAc–AcOH buffer, pH 4.0	45 °C	24 h	2–50 oligomers	[100]

**Table 2:** Degradation of chitin and chitosan via physical methods.

Methods	Chemical Concentration	T (°C)	Time	% MW Reduction	Ref.
Microwave 400 W	2% acetic acid	N/A	25 min	79%	[101]
Microwave 100 W	0.1 M acetic acid	N/A	20 min	92.5%	[87]
Ultraviolet 1 kW power	0.1 M acetic acid	25	15 min	98.5%	[88]
Ultrasonication 200 W	0.1 M acetic acid	N/A	120 min	52%	[88]
^60^Co γ-rays Radiation	2% acetic acid mixed 10 mL hydrogen peroxide	N/A	7 h	95%	[102]
Impinging stream and jet cavitation	acetic acid mixed with sodium acetate trihydrate	40	30 min	88%	[103]
Hydrodynamic cavitation	0.2 acetic acid mixed with 0.1 sodium acetate	40	30 min	95%	[104]
Plasma 350 W	1% acetic acid	N/A	180 min	83 %	[105]
SPP	1 M acetic acid	25–30	300 min	n/a	[46]
SPP	1 M acetic acid	25–30	300 min	96%	[40]
SPP	0.1 M acetic acid 4 M hydrogen peroxide	Room temperature	60 min	85%	[74]
SPP	0.00155 mM carboxylic acids	Room temperature	60 min	86%	[106]
SPP	0.02 M sodium chloride	Room temperature	60 min	96%	[39]

## Data Availability

All data will be made available upon request.

## Supplementary Materials

The following are available online at https://www.mdpi.com/article/10.3390/ijms22094308/s1.
