# Supplementary material for: Insight on Solution Plasma in Aqueous Solution and Their Application in Modification of Chitin and Chitosan
Source: Int J Mol Sci. 2021 Apr 21;22(9):4308. doi: 10.3390/ijms22094308 (PMC8122608; doi:10.3390/ijms22094308)
Supplement: Supplementary file 1 [file ijms-22-04308-s001.zip › ijms-1183128-supplementary.pdf]

Publications	Year	Raw materials
J.-J. Zou et al. / Carbohydrate Polymers 55 (2004) 23	2004	Starch
I. Prasertsung et al. / Carbohydrate Polymers 87 (2012) 2745	2012	Chitosan
Y. Wen et al. / Journal of Hazardous Materials 201– 202 (2012) 162	2012	Chitosan
Fengming Ma, Int. J. Mol. Sci. 2012, 13, 7788-7797; doi:10.3390/ijms13067788	2012	Chitosan
A. Watthanaphanit, N. Saito / Polymer Degradation and Stability 98 (2013) 1072	2013	Sodium algenate 0.2%
I. Prasertsung et al. / Polymer Degradation and Stability 98 (2013) 2089	2013	Chitosan
O. Pornsunthorntawee et al. / Carbohydrate Polymers 102 (2014) 504	2014	Chitosan
A. Watthanaphanit et al. / Journal of the Taiwan Institute of Chemical Engineers 45 (2014) 3099	2014	Alginate
L. Na et al./ Plasma Science and Technology 16 (2014) 128	2014	Chitosan
F. Syahrial et al./ Journal of the Japan Institute of Energy 93 (2014)1207	2014	Glucose and Cellulose (saccharide)
R. Molina et al./ Cellulose 21 (2014) 729	2014	Cellulose
T. Tantiplapol et al. / Innovative Food Science and Emerging Technologies 32 (2015) 116	2015	Chitosan
M. Davoodbasha et al./ RSC Advance 5 (2015) 35052	2015	Cellulose
I. Rahim et al./Journal of Power and Energy Engineering 3 (2015) 28	2015	Cellulose
M. Davoodbasha et al. / Archives of Biochemistry and Biophysics 605 (2016) 49	2016	Chitosan
N. Janpetch et al. / Carbohydrate Polymers 148 (2016) 335	2016	Baterial cellulose
VA Titov et al./ High Energy Chemistry 50 (2016) 411	2016	Chitosan
D. Nikitin et al. / Carbohydrate Polymers 154 (2016) 30	2016	Chitosan
K. Tange et al./ Journal of the Japan Institute of Energy 95 (2016) 1105	2016	Cellulose
I. Prasertsung et al. / Carbohydrate Polymers 172 (2017) 230	2017	Microcrystalline cellulose powder
F. Ma et al. / International Journal of Biological Macromolecules 98 (2017) 201	2017	Chitosan
F. Ma et al. / International Journal of Biological Macromolecules 103 (2017) 501	2017	Chitosan
C. Chokradjaroen et al. / Carbohydrate Polymers 167 (2017) 1	2017	Chitosan
Y. Sun et al. / Carbohydrate Polymers 164 (2017) 222	2017	Chitosan
Y. Sun et al./ Environmental Technology 40 (2019) 954	2017	Chitosan andacrylamide
F Syahrial et al./, Journal of the Japan Institute of Energy 96 (2017) 451	2017	Cellulose
J. Wu et al./ International Journal of Biological Macromolecules 117 (2018) 1299	2018	Auricularia auricula polysaccharide
F. Ma et al./ Carbohydrate Polymers 198 (2018) 575	2018	Auricularia auricula polysaccharide
Chokradjaroen et al./ Japanese Journal Applied Physics 57 (2018) 0102B5	2018	Chitosan

M. Davoodbasha et al. / International Journal of Biological Macromolecules 118 (2018) 1511	2018	Chitosan
C. Chokradjaroen et al./ Carbohydrate Polymers 201 (2018) 20	2018	Chitosan powder
D.M. Panaitescu et al./ Nanomaterials 8(2018) 467	2018	nanocellulose
S. Vizireanu et al./ Scientific Reports 8 (2018) 15473	2018	Cellulose
F. Ma et al./Carbohydrate Polymers 257 (2021) 117567	2019	Chitosan
I. Prasertsung et al./Carbohydrate Polymers 205 (2019) 472	2019	Cassava starch waste
T. Ju et al./ ACS Sustainable Chem. Eng. 7(2019)593	2019	Gracilaria lemaneiformis (medicinal mushroom)
K. Tange, Journal of the Japan Institute of Energy 98 (2019)265	2019	Cellulose
Y. Zhou et al. / Poymers 11 (2019) 8	2019	Waxy and Normal Maize Starch
R. Rujiravanit et al./ Carbohydrate Polymers 228 (2020) 115377	2020	Chitin
V. Titov et al./ Materials 13 (2020) 4821	2020	Chitosan
C. Chokradjaroen et al./ Carbohydrate Polymers 237 (2020) 116162	2020	CMC-AuNPs
B. Honnorat et al./ AIP Advances 10 (2020) 095025	2020	Sodium carboxymethyl cellulose
O. L. H. Li,et al./ Int. J. Plasma Environ. Sci. Technol. 14 (2020) e01005	2020	Cellulose
